# Scapular dyskinesis in myotonic dystrophy type 1: clinical characteristics and genetic investigations

**DOI:** 10.1007/s00415-019-09494-8

**Published:** 2019-08-31

**Authors:** N. C. Voermans, R. C. van der Bilt, J. IJspeert, J. Y. Hogrel, M. Jeanpierre, A. Behin, P. Laforet, T. Stojkovic, B. G. van Engelen, G. W. Padberg, S. Sacconi, R. J. L. F. Lemmers, S. M. van der Maarel, B. Eymard, G. Bassez

**Affiliations:** 1 Department of Neurology, Donders Institute for Brain, Cognition and Behaviour, Radboud University Medical Center, Nijmegen, The Netherlands; 2 Department of Rehabilitation, Donders Institute for Brain, Cognition and Behaviour, Radboud University Medical Center, Radboudumc, PO Box 9101, 6500 HB Nijmegen, The Netherlands; 3 AP-HP; Centre de référence des maladies neuromusculaires Nord-Est-Ile de France, Institut de Myology, Paris, France; 4 AP-HP, Hôpital Cochin, Paris, France; 5 Neurology Department, Raymond Poincaré Teaching Hospital, Nord/Est/Ile de France Neuromuscular Center, AP-HP Garches, Garches, France; 6 INSERM U1179, END-ICAP, Université Versailles Saint-Quentin-en-Yvelines, Montigny-le-Bretonneux, France; 7 Université Côte d’Azur, Peripheral Nervous System, Centre Hospitalier Universitaire de Nice, Muscle and ALS department, Nice, France; 8 Inserm CNRS, Institute for Research on Cancer and Aging of Nice (IRCAN), Université Côte d’Azur, Nice, France; 9 Department of Human Genetics, Leiden University Medical Center, Leiden, The Netherlands

**Keywords:** DM1, Myotonic dystrophy, Scapular dyskinesis, Scapular winging, Shoulder weakness, FSHD

## Abstract

**Objective:**

To study scapular winging or other forms of scapular dyskinesis (condition of alteration of the normal position and motion of the scapula) in myotonic dystrophy type 1 (DM1), which is generally considered to be a distal myopathy, we performed an observational cohort study.

**Methods:**

We performed a prospective cohort study on the clinical features and progression over time of 33 patients with DM1 and pronounced, mostly asymmetric scapular winging or other forms of scapular dyskinesis. We also explored if scapular dyskinesis in DM1 has the same genetic background as in facioscapulohumeral muscular dystrophy type 1 (FSHD1).

**Results:**

The cohort included patients with congenital (*n* = 3), infantile (*n* = 6) and adult-onset DM1 (*n* = 24). Scapular girdle examination showed moderate shoulder girdle weakness (mean MRC 3) and atrophy of trapezius, infraspinatus, and rhomboid major, seemingly similar as in FSHD1. Shoulder abduction and forward flexion were limited (50–70°). In five patients, scapular dyskinesis was the initial disease symptom; in the others it appeared 1–24 years after disease onset. Follow-up data were available in 29 patients (mean 8 years) and showed mild to severe increase of scapular dyskinesis over time. In only three patients, DM1 coexisted with a FSHD mutation. In all other patients, FSHD was genetically excluded. DM2 was genetically excluded in nine patients. The clinical features of the patients with both DM1 and FSHD1 mutations were similar to those with DM1 only.

**Conclusion:**

Scapular dyskinesis can be considered to be part of DM1 in a small proportion of patients. In spite of the clinical overlap, FSHD can explain scapular dyskinesis only in a small minority. This study is expected to improve the recognition of shoulder girdle involvement in DM1, which will contribute to the management of these patients.

**Electronic supplementary material:**

The online version of this article (10.1007/s00415-019-09494-8) contains supplementary material, which is available to authorized users.

## Introduction

Muscle weakness in myotonic dystrophy type 1 (DM1) typically occurs in distal upper and lower limbs, neck and face muscles. Bulbar and respiratory muscle weakness evolves gradually. Proximal muscle weakness in the arms or shoulder girdle weakness may arise later in the disease and generally remains less pronounced than distal muscle weakness. Early pronounced involvement of the proximal muscles in DM1 has only rarely been reported [1–4].

Similarly, scapular winging has received only little attention in DM1 [5–7]. Scapular winging is one of the manifestations of scapular dyskinesis which refers to the alteration of the position and motion of the scapula, secondary to weakness or discoordination of the serratus anterior muscle and other scapular fixators [8]. It thus points to a scapular dyskinetic motor profile and not to a movement disorder caused by a central nervous system disease. This dyskinesis results in an abnormal scapulohumeral rhythm in all three planes (Supplemental Fig. 1 and 2). Hamano et al. reported two members of the same family with adult-onset DM1 and scapular winging. On muscle MRI, marked atrophy of the serratus anterior and latissimus dorsi muscle was seen in both [5]. In the patient reported by Masciullo et al., scapular winging was associated with the coexistence of FSHD1 [6]. In the fourth patient, DM1 was suspected because of the positive family history in spite of the atypical presentation with scapular winging [7]. These reports call for further research into the prevalence, features and pathophysiology of scapular dyskinesis in DM1.

We, therefore, performed a prospective cohort study of 33 patients with DM1 and pronounced, mostly asymmetric, abnormal scapular position and motion with more pronounced proximal than distal weakness. We systematically assessed DM1 severity and characteristics, shoulder girdle strength, maximal range of shoulder abduction and forward flexion, and evaluated the progression over time. The type of scapular dyskinesis was retrospectively classified [8]. All patients were genetically tested for FSHD1, nine were tested for myotonic dystrophy type 2 (DM2), and in seven patients, the D4Z4 methylation was assessed, with subsequent sequencing of *SMCHD1* when appropriate. This study is expected to improve the recognition and understanding of shoulder girdle involvement in DM1, which eventually will contribute to better management of these patients.

## Methods

### Patients

In the last years, we paid attention to overt shoulder involvement occurring in DM1 patients at the outpatient departments of the Institute of Myology in Paris, France and the Radboudumc in Nijmegen, the Netherlands. The total cohort of DM1 patients followed up in these centres was approximately 900 and 300 patients, respectively. We systematically assessed all DM1 patients with known scapular dyskinesis. The study was performed according to the guidelines of the local ethical committees, and informed consent was obtained from all patients for use of the photographs and videos.

Patients meeting the following inclusion criteria were included:

(1) DM1 confirmed by genetic testing; (2) more pronounced scapular girdle weakness (modified Medical Research Council (MRC) ≤ 3 and inability to perform the whole range of motion against gravity) than distal muscle weakness in upper and lower limbs (MRC > 3 and ability to perform the whole range of motion against gravity); (3) moderate-to-severe scapular dyskinesis (alteration of the normal position and motion of the scapula) described by two neurologists specialized in neuromuscular diseases (BE and NV) and confirmed by a physical therapist specialized in scapular dyskinesis on photographs and videos (JIJ); (4) ambulatory at onset of scapular girdle weakness; and (5) no other explanation for scapular dyskinesis other than weakness and/or discoordination of the scapular fixator muscles.

### Clinical evaluation

#### DM1 disease severity and features

CTG expansion length and DM1 type (congenital, infantile, juvenile, adult onset) were extracted from the medical record. Muscle strength was assessed by MRC measurement of elbow flexors and extensors, wrist flexors and extensors, finger flexors and extensors, hip flexion, extension, abduction, and adduction, knee flexors and extensors, and foot dorsiflexion and plantar flexion. DM1 disease severity was rated by the muscular impairment rating scale (MIRS) [9]: (1) no muscular impairment; (2) minimal signs; myotonia, jaw and temporal wasting, facial weakness, neck flexor weakness, ptosis, nasal speech, no distal weakness except isolated digit flexor weakness; (3) distal weakness; no proximal weakness except isolated elbow extensor weakness; (4) mild-to-moderate proximal weakness (MRC scale 4); and (5) severe proximal weakness (MRC scale ≤ 3). We added a modified MIRS score: the ‘MIRS lower limb (LL)’ to rule out the effect of shoulder girdle weakness, whose presence automatically results in a MIRS score of at least four due to proximal muscle involvement.

The presence of facial muscle weakness, dysarthria, dysphagia, cervical weakness (neck flexors and extensors MRC scale ≤ 4), stepping gate, myotonia, and hypersomnia was assessed as dichotomous outcome: present or absent. Functional ability was tested with the 10-m walk test and the Walton scale. Data on respiratory muscle involvement (vital capacity and use of non-invasive ventilation) and cardiac muscle involvement (cardiac conduction defect and arrhythmia, pacemaker implantation), cataracts and mental retardation were collected. In addition, CK levels were noted.

#### Shoulder girdle strength and muscle mass

Clinical evaluation of shoulder girdle strength included manual muscle testing of shoulder girdle muscles using the MRC during shoulder abduction and forward flexion and range of maximal shoulder abduction and forward flexion (simultaneously tested at both sides). Furthermore, muscle mass of trapezius descendens and infraspinatus was evaluated qualitatively (atrophy or no atrophy). Progression over time was evaluated by repetitive evaluations and subsequent visits to the outpatients department and photographs. We calculated the loss of shoulder abduction and forward flexion per year by taken the average loss of degrees of movements for left and right and divide by the number of years of follow-up.

#### Scapular dyskinesis classification

Normal scapular movement was defined as follows: the scapula normally moves over three axes continuously (Supplemental Fig. 1). During normal scapular movement, the scapula follows the arm in elevation and abduction in upward rotation, posterior tilt and internal rotation. When the arm moves downward again, the scapula follows in downward rotation, external rotation and anterior tilt [10]. Scapular dyskinesis was defined as an alteration in the normal position or motion of the scapula during coupled scapulohumeral movements, and was classified as prominence of the inferomedial border of the scapula (type 1; tipping); prominence of the entire medial border (type 2; winging), or prominence of the superomedial border (type 3; superior translation) (Supplemental Fig. 2) [8]. The term shoulder dyskinesis does not imply a cause in the peripheral or central nervous system; it reflects the functional disturbance occurring as a combination of shoulder girdle muscle weakness and altered coordination of shoulder girdle movements. The shoulder dyskinesis was retrospectively assessed in patients of whom sufficient photographs (and videos) were taken during consultations at onset and during follow-up by two neurologists and an experienced shoulder physical therapist (JIJ). Right and left shoulders were separately classified as type 1, 2 or 3 dyskinesis.

### Genetic analysis of FSHD and DM2

Genetic testing for FSHD1 and DM2 was performed in Paris and Marseille, France and in Leiden, the Netherlands. In addition, blood samples of seven patients were sent from France to Leiden in 2017 for assessment of D4Z4 methylation as previously reported. This included testing for the presence of a permissive haplotype [11]. Subsequent genetic testing on FSHD2 was performed if appropriate [12, 13].

### Data analysis

Qualitative variables are expressed as number (*n*) and percentage (%), and quantitative variables as means, standard deviation (SD) and range. For the comparison between groups, we used the unpaired *t* test.

### Data availability statement

Anonymized data of individual patients will be shared by request from any qualified investigator.

## Results

### Patients

We identified 44 patients with scapular dyskinesis of which 11 were excluded for the following reasons. Five patients had a limitation of shoulder abduction and atrophy of the shoulder muscles but without scapular dyskinesis, two patients had more pronounced distal than proximal muscle weakness, in three patients the scapular dyskinesis and limitation of arm elevation could be explained by axial muscle weakness rather than of weakness of the scapula fixators, and in one patient, DM1 was not genetically confirmed. Hence, we included 33 patients from 29 families (30 at the Institute of Myology and 3 at the Radboudumc). A description of the patient characteristics is shown in Table 1. Clinical follow-up data were available in 29 patients (mean duration of follow-up of 8 years, with 2–12 visits), and images (photographs or videos) in 21 patients. Supplemental Table 2 shows the findings of subsequent visits in individual patients.

**Table 1:** Patient characteristics and DM1 disease severity

	*n*	Mean ± SD [range]/number (%)
Age at last evaluation (years)	33	48.1 ± 11.6 [23–69]
Sex	33	
Male		25 (76)
Female		8 (24)
CTG expansion (length)	33	679 ± 312 [93–1500]
Follow-up in years	33	8.3 ± 4.6 [0–24]
Type of DM1:	33	
Congenital		3 (9)
Infantile		3 (9)
Juvenile		2 (6)
Adult onset		23 (70)
Late onset		2 (6)
MIRS LL at last evaluation	31	4.2 ± 0.6 [2–5] MIRS 2: *n* = 1 MIRS 3: *n* = 2 MIRS 4: *n* = 17 MIRS 5: *n* = 11
MIRS LL at onset scapular dyskinesis	24	3.5 ± 0.5 [3–4]
Facial weakness	33	32 (97)
Symmetrical		31 (94)
Asymmetrical		1 (3)
Dysarthria	33	16 (48)
Dysphagia	33	12 (36)
Cervical weakness	33	32 (97)
Stepping gate	33	29 (88)
Myotonia	33	28 (85)
10-m walk test (s)	28	18.2 ± 10.0 (7 – 120)
Walton score	26	4.2 ± 1.4 (3 – 7)
Vital capacity (in % for age, sex and length)	30	68 ± 17 (34 – 104)
Non-invasive ventilation	32	
Yes		15 (47)
No		17 (53)
Hypersomnia	32	7 (22)
Cardiac involvement	33	
Conduction defect		6 (18)
Arrhythmia		4 (12)
Pacemaker implantation		10 (30)
No involvement		23 (70)
Cataract	31	21 (68)
Mental retardation	33	3 (9)
CK level (U/l)	29	246 ± 136 (80 – 686)

Data presented as mean ± SD [range] or *n* (%)

The cohort included patients with congenital (*n* = 3), infantile (*n* = 3), juvenile (*n* = 2), adult-onset DM1 (*n* = 23), and late onset (*n* = 2). It included four families with two members with scapular dyskinesis. Three of them consisted of two affected brothers. In one of these three families, three other siblings with DM1 had no shoulder girdle weakness or scapular dyskinesis. The fourth family consisted of two affected cousins with a congenital and an infantile form of DM1. Their mothers, who are sisters, had DM1 but without scapular dyskinesis. One patient was initially diagnosed with both DM1 and FSHD by a neurologist specialized in neuromuscular diseases, and FSHD was later excluded by genetic testing.

### Clinical features

#### DM1 features and disease severity

Clinical features of DM1, including MIRS, respiratory and cardiac involvement and functional abilities are shown in Table 1. The MIRS pointed to mild-to-moderate proximal weakness in most patients at last evaluation (mean MIRS 4.2; MIRS 2: *n* = 1; MIRS 3: *n* = 2; MIRS 4; *n* = 17 and MIRS 5: *n* = 11; not available: *n* = 2). No difference could be seen between the MIRS and the MIRS modified for only lower limbs. We observed symmetrical facial weakness in 97%, dysarthria in 48%, dysphagia in 36%, respiratory muscle weakness for which non-invasive ventilation was required in 47%, and cardiac involvement leading to pacemaker use in 30%. Data of individual patients, including results of MRC muscle strength measurement of distal arm and lower limb muscles, are shown in Supplemental Table 1. Sensory disturbances and fasciculations were not observed.

The three patients with both DM1 and FSHD1 mutations (patients 1–3; Supplemental Table 1) had similar clinical DM1 features compared to the patients without a FSHD1 mutation. One patient had an involvement of the respiratory and cardiac muscles.

#### Shoulder girdle strength and muscle mass

The results of assessment are shown in Table 2. The mean age of onset of shoulder girdle weakness was 26.9 ± 10.8 years. The mean muscle strength of the shoulder girdle was MRC 3, varying between MRC 2 and 5 (at least unilaterally < 3). The mean maximal degree of shoulder abduction was 70/60 (right/left) and of shoulder forward flexion 60/50 (right/left), but both with a large range of 0°–180° (at least unilaterally reduced motion) (Table 2 and Supplemental Table 2).

**Table 2:** Shoulder girdle weakness and scapular dyskinesia

Shoulder girdle weakness: maximal abduction and anteflexion (*n* = 33)	
Age of onset of shoulder girdle weakness (mean age in years ± SD [range])	26.9 ± 10.8 [0–52]
Degree of maximal shoulder abduction at last evaluation (mean range in degrees ± SD [range])	70/60 ± 40/35 [0–180]
Degree of maximal shoulder anteflexion at last evaluation (mean range in degrees ± SD [range])	60/50 ± 28/28 [20/0–180]
Shoulder girdle atrophy (*n* = 15-6) (%)	
Atrophy of trapezius descendens (*n* = 15)	12 (80)
Atrophy of infraspinatus (*n* = 16)	6 (38)
Scapular dyskinesis (*n* = 33) (%)	
Age-onset scapular dyskinesis (mean age in years ± SD [range])	37.9 ± 11.5 [6–61]
Scapular dyskinesis characteristics	
Unilateral	10 (30)
Bilateral	23 (70)
Symmetrical	11 (33)
Asymmetrical	22 (67)
Left	6 (27)
Right	15 (68)
Unknown	1 (5)

	Right	Left
Scapular dyskinesis classification (based on retrospective visual evaluation of photographs) (*n* = 21)		
Type 1: prominence of the inferomedial border of the scapula (tipping)	1 (5)	1 (5)
Type 2: prominence of the entire medial border (winging)	7 (33)	3 (14)
Type 3: prominence of the superomedial border (superior translation)	11 (34)	16 (76)
No dyskinesis	0 (0)	1 (5)

Data presented as mean ± SD [range] or *n* (%)

**Fig. 1:**
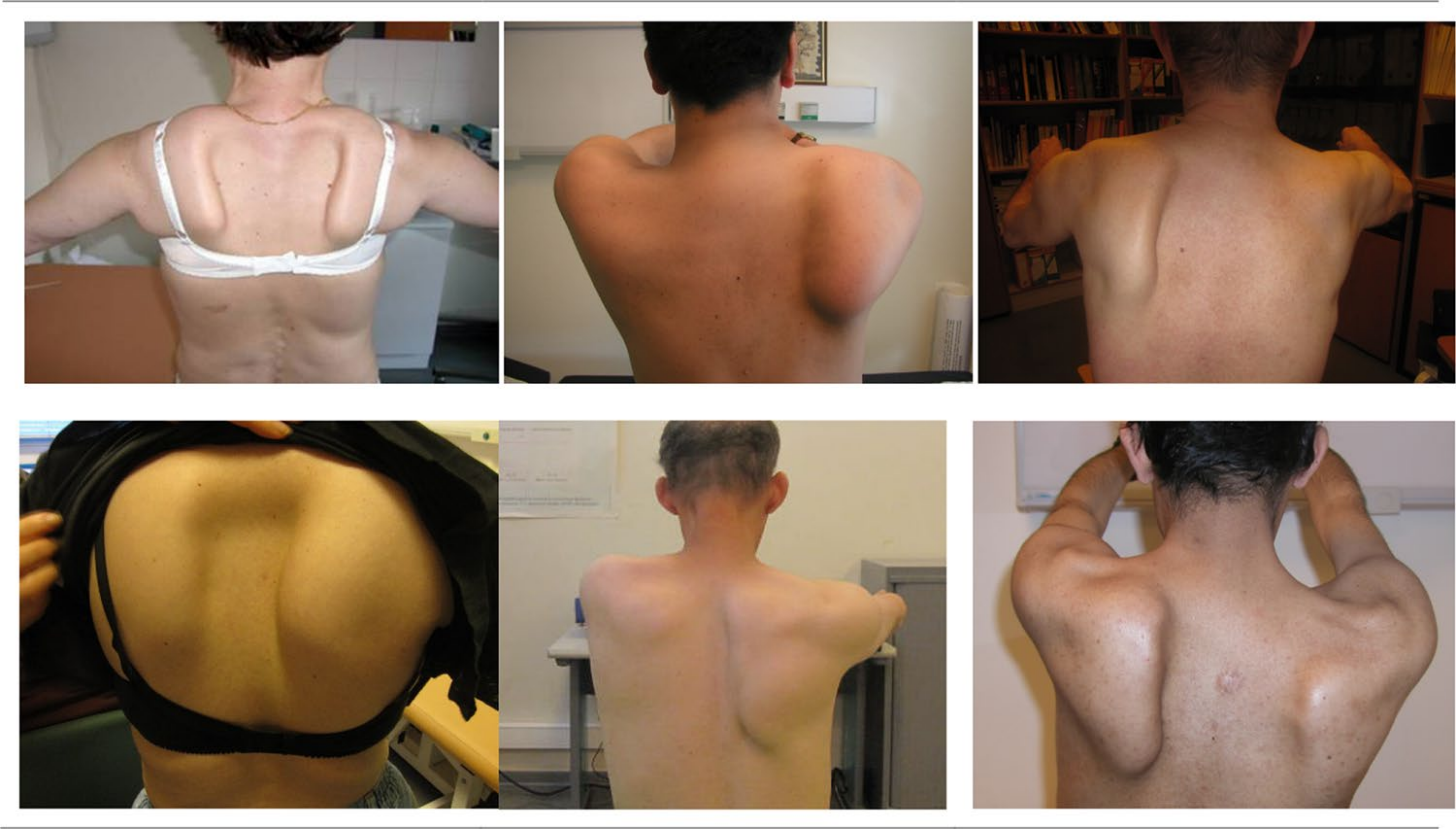
Six patients with shoulder girdle weakness and scapular dyskinesis. Upper row—left image (patient 10): age 65 years, adult form DM1, onset scapular dyskinesis at age 12 years, D4Z4 repeat of 9 on chromosome 4qA, SD type 3 on both sides, overactivation of rhomboideus minor muscles. Middle image (patient 25): age 35 years, adult form DM1, onset scapular dyskinesis at age 10 years, FSHD negative, SD type 3 on the right side. Right image (patient 6) age 54 years, adult form DM1, onset scapular dyskinesis at age 48 years, FSHD negative, SD type 3 on the left side. Bottom row—left image (patient 11): age 21 years, congenital form DM1, onset scapular dyskinesis at age 20 years, FSHD not tested SD type 3 on both sides, severity R > L, rhomboid minor over active. Middle image (patient 31): age 30 years, congenital form DM1, onset scapular dyskinesis at age 20 years, FSHD not tested, Sd type 2 on the right side. Right image (patient 29): age 28 years, infantile form DM1, onset scapular dyskinesis at age 26 years, FSHD negative, SD type 1 on the right side and type 3 on the left side

Besides involvement of the scapula fixator muscles, the other muscles of the scapular girdle showed atrophy: 80% of the patients had atrophy (symmetrical or asymmetrical) of the trapezius and 38% showed atrophy of the infraspinatus. Figure 1 shows six patients with different patterns of shoulder girdle weakness and scapular dyskinesis.

The mean onset of scapular dyskinesis was at age 38 years, 11 years after the mean onset of shoulder girdle muscle weakness. However, in five patients, scapular dyskinesis was the initial symptom: patient 2 (female, both DM1 and FSHD1, 46 years at presentation, asymmetrical), patient 6 (male, 34 years, symmetrical), patient 16 (male, 36 years, symmetrical), patient 21 (male, 15 years; asymmetrical), and patient 22 (female, 41 years; symmetrical)).

In the two other patients with both DM1 and FSHD1 mutations (patients 1 and 3), scapular dyskinesis appeared 14 and 21 years after the onset of muscle weakness.

#### Scapular dyskinesis and follow-up

Scapular dyskinesis classification could be assessed in 21 patients based on retrospective evaluation of photographs and/or videos. It was mostly bilateral (70%) and asymmetrical (67%) with a dominant involvement of the right side (Table 2 and Supplemental Table 2). Most patients showed a type 3 dyskinesis [superior translation; 11/21 (52%) on right and 16/21 (76%) on left side], some with overactivation of rhomboids, levator scapulae and minor pectoral muscles, which will probably increase the tendency for downward rotation of the scapula.

**Fig. 2:**
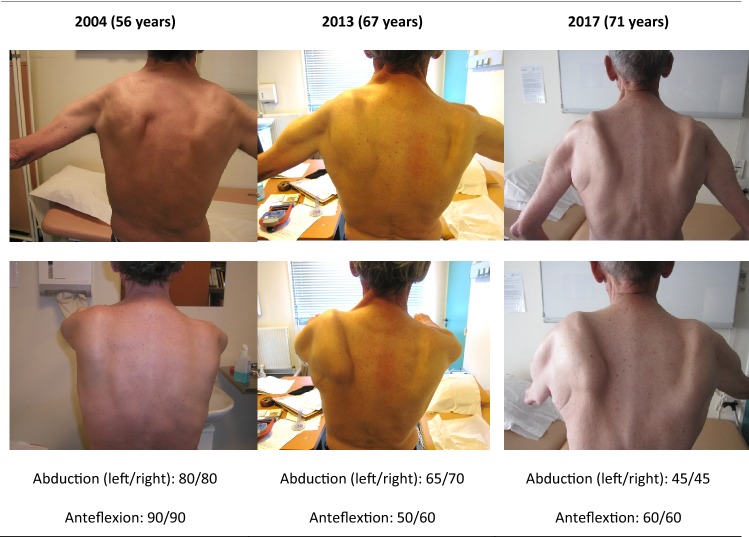
Patient 17 during follow of 13 years. Patient 17 with adult form DM1, onset scapular dyskinesia at age 54 years, FSHD negative, with range of maximal motion in degrees

**Fig. 3:**
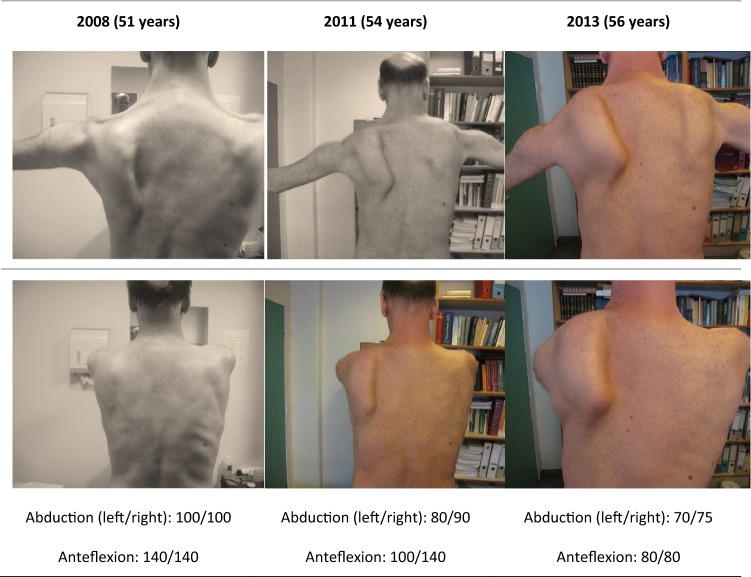
Patient 8 during follow-up of 10 years. Patient 8 with adult form of DM1, onset scapular dyskinesia at age 51 years, FSHD negative, with range of maximal motion in degrees

Follow-up data of shoulder girdle weakness and scapular dyskinesis were available in 29 of the 33 patients with an overall mean follow-up of 8 years. All patients showed an increase of the scapular dyskinesis over time (Table 2; Supplemental Tables 1 and 2; Figs. 1, 2, 3). This progression was clearly captured on subsequent photographs, of which two examples are shown in Figs. 2 and 3. In three out of the ten patients with unilateral scapular dyskinesis at onset, the scapular dyskinesis became bilateral during the course of the disease. Furthermore, a decrease in the degree of shoulder movements, forward flexion and abduction, was seen in all 29 patients with follow-up. In 12 patients, this was a rapid decline from normal range of movement (forward flexion and abduction of 180°) to severe shoulder disability (forward flexion and abduction of 20°–50°) in less than 10 years. The mean loss of forward flexion and abduction was 10.3° and 6.3° per year.

### Genetic analysis of FSHD and DM2

Genetic testing for FSHD1 was performed in Paris, France (*n* = 24) and in Leiden, the Netherlands (*n* = 12) additional testing for the presence of a permissive haplotype was performed in 11 patients, and methylation levels were assessed in 7 patients. A contraction of the D4Z4 repeat to 1–10 units on the permissive 4qA haplotype, indicating FSHD1, was detected in three patients. They had a contraction of the D4Z4 repeat of 8, 9 and 10 units, respectively (patients 1–3). In four patients, FSHD1 testing showed a D4Z4 repeat of 11 units (patients 4–7). Blood samples of six patients were analyzed in Leiden for FSHD2 and for changes in methylation by Southern blotting and methylation-sensitive restriction enzyme FseI [13]. For one sample (patient 5; − 35%), we found delta1 methylation value of − 35%, which is far below the threshold for FSHD2 (− 21%). However, *SMCHD1* Sanger sequencing did not reveal any potential pathogenic variants and the shortest permissive allele was much longer (74 units) than the typical repeat size found in FSHD2 patients (8–20 units) [14]. Furthermore, detailed Southern blot analysis did not reveal an FSHD2-associated D4Z4 duplication in him (patient 5) [15] DM2 was genetically excluded in nine patients (Supplemental Table 1).

## Discussion

We here report the clinical features and progression over time of 33 patients with DM1 and pronounced, mostly asymmetrical scapular dyskinesis resulting from shoulder girdle weakness or discoordination of the scapular fixator muscles or both. This occurred in all subtypes of DM1 (from congenital to late onset), either as an early manifestation or later in the disease course. Genetic studies showed a concomitant FSHD1 mutation (D4Z4 repeat of 8–10 units) in 3 of 33 patients, and reduced methylation of the D4Z4 repeat (delta1: − 35) in 1 of 9 patients tested; however, in absence of a *SMCHDH1* mutation, therefore, excluding FSHD2. We conclude that shoulder girdle weakness with scapular dyskinesis is an inherent feature of DM1 in a small subset of patients. This study thus confirms the previous reports in single cases [5, 7].

The DM1 patients with scapular dyskinesis were mildly to severely affected (MIRS 2: *n* = 1; MIRS 3: *n* = 2; MIRS 4: *n* = 17; MIRS 5: *n* = 11) and had otherwise typical DM1 features. The mean MIRS score at last evaluation was 4.2. We observed symmetrical facial weakness in 97%, dysarthria in 48%, respiratory muscle weakness for which non-invasive ventilation was required in 47%, and cardiac conduction defects leading to pacemaker use in 30%. Overall, these severity parameters are above the mean severity scores reported in an adult DM1 population (and in the registry cohort) [16].

The retrospective evaluation of successive photographs and videos of 21 patients shed light on the abnormal position and motion of the scapula. Most patients showed a type 3 scapular dyskinesis (superior translation), some with overactivation of rhomboids, levator scapulae and minor pectoral muscles, which is likely to increase the tendency for anterior tilt, downward rotation, and internal rotation of the scapula. Recruitment of serratus anterior and trapezius descendens seemed very limited and decreased over time, disabling the scapula in stabilizing in posterior tilt, upward rotation and external rotation. This subsequently causes an increase in scapular dyskinesis. This pattern is very similar to our observations in FSHD patients. The scapular dyskinesis was in all cases associated with a progressive reduction of range of motion of shoulder abduction and forward flexion.

The systematic assessment of shoulder girdle strength and function which has been performed in IM from 2000 to 2017 enables a rough speculation of the scapular dyskinesis in DM1: 30 patients among a population of 900 patients suggests a prevalence of 3%. This might be an underestimation since only patients with more proximal than distal weakness were included, whereas scapular dyskinesis may also occur in patients with pronounced distal weakness. Furthermore, patients with scapular dyskinesis type 1 with still shoulder abduction MRC > 3 were not included either. The three cases detected in the Netherlands in 2015–2017 confirm the existence of this manifestation in another population.

The recognition of this DM1 phenotype, although rare, is important for timely management since clinicians might neglect the testing of muscles that are not considered to be typically involved in a specific disease. Similarly, patients might fail to report the limitation in shoulder movements since they consider it part of their muscle disease. Furthermore, one patient was initially misdiagnosed with FSHD by a neurologist specialized in neuromuscular diseases. Only after genetic testing, FSHD was excluded leading to consider the DM1 diagnosis. In five patients the scapular dyskinesis was the presenting symptom, and in all other patients it developed after DM1 was diagnosed, with a mean disease duration of 11 years. This underlines the importance of shoulder girdle function testing in all DM1 patients on annual checkup.

Patients with winging report diffuse neck, shoulder girdle, and upper back pain, which may be debilitating, associated with abduction and overhead activities [17]. First, it is important to differentiate between patients that show scapular dyskinesis as a consequence of muscle weakness and patients that show disorganization of activation in scapular stabilizers. The latter group may be treated with motor control therapy, which would enable them to relearn correct activation patterns [18]. This training is expected to improve the stabilization of the scapula on the thoracic wall and possibly protecting the re-activated musculature against accelerated dystrophy due to inactivity. Furthermore, scapular dyskinesis due to muscle weakness of the scapular fixators is most obvious during active movements and is usually absent at rest [19]. Furthermore, decreased shoulder abduction and forward flexion can also be caused by weakness of the axial muscles. In these patients presenting with camptocormia, symmetrical scapular dyskinesis and a limitation of shoulder abduction and forward flexion, support of paraspinal muscles (by lying down or sitting straight on a chair with backrest) reduces the limitations in shoulder movements and resolves scapular dyskinesis. This makes postural therapy a viable option to explore in further research.

At the onset of this study, we had different hypothesis on the causes of shoulder girdle involvement. The first hypothesis, coexistence of DM1 and FSHD1, was based on the fact that the two diseases are the two most common inherited myopathies in adulthood and were simultaneously detected in one of the previous case reports [6]. We tested all patients, and only in three patients (1, 2 and 3; 9%) FSHD1 was genetically confirmed. Remarkably, all three had a D4Z4 repeat size (8–10 units) that is found in the normal population with a frequency of 1–3% [20, 21]. Therefore, this finding might as well be coincidental. A second hypothesis, coexistence of DM1 and DM2 was also rejected based on negative genetic testing for DM2 in nine patients.

To look for other explanations and, additional genetic analysis was performed based on the clinical similarity with FSHD. FSHD2, which is the cause of FSHD in less than 5% of the patients [13], was excluded by D4Z4 CpG methylation analysis in eight out of nine patients. In only one patient, we found D4Z4 hypomethylation and a permissive 4qA allele, but without identifying a pathogenic SMCHD1 variant [14]. Hence, the hypothesis of a FSHD2 mutation or D4Z4 hypomethylation otherwise as explanation for scapular dyskinesis in DM1 seems unlikely based on these results, but remains to be rejected in a larger group of patients. Together, these findings suggest that the molecular mechanism is independent of the known FSHD pathophysiology in most patients.

There are a number of limitations. First, DNA samples were unavailable in four patients. This limited the screening for FSHD1 and 2, and DM2, which should be part of diagnostic analysis in all DM1 patients with pronounced shoulder girdle weakness or scapular dyskinesis who do not have a D4Z4 repeat contraction. Second, we have taken only subsequent photographs in most patients, while videos would have enabled a more accurate assessment of scapular dyskinesis. Our preferred next research step would, therefore, be a comparative trial on the effect of targeted physical therapy on scapular dyskinesis in both DM1 and FSHD patients, including quantitative clinical assessment and imaging (MRI or muscle ultrasound) before and after treatment [22, 23]. Furthermore, such study will allow estimating the prevalence of this condition.

In short, this study demonstrates that scapular dyskinesis resulting from shoulder girdle weakness or altered coordination or both occurs in a subgroup of DM1 patients. Although coexistence of FSHD1 needs to be excluded, scapular dyskinesis is inherent to DM1 in most patients, independent of the DM1 type. This knowledge is essential for adequate management.

### Electronic supplementary material

Below is the link to the electronic supplementary material.

Supplementary material 1 (PPTX 2036 kb)

Supplementary material 2 (XLSX 88 kb)

## Appendix 1

See Table 3.

**Table 3:** Contributions

Name	Location	Role	Contribution			
			DoS	AoD	IoD	DoM
N. C. Voermans	Radboudumc Nijmegen, Nl Nicol.Voermans@radboudumc.nl	Author	x	x	x	x
R. C. van der Bilt	Radboudumc Nijmegen, Nl roosmarijnvanderbilt@hotmail.com	Author	x	x	x	x
J. IJspeert	Radboudumc Nijmegen, Nl Jos.IJspeert@radboudumc.nl	Author			x	x
J. Y. Hogrel	Institute Myologie, Paris, F jy.hogrel@institut-myologie.org	Author		x		x
M. Jeanpierre	Institute Myologie, Paris, F marc.jeanpierre@aphp.fr	Author		x		x
A. Behin	Institute Myologie, Paris, F anthony.behin@aphp.fr	Author		x		x
P. Laforet	Institute Myologie, Paris, F pascal.laforet@aphp.fr	Author		x		x
T. Stojkovic	Institute Myologie, Paris, F tanya.stojkovic@aphp.fr	Author		x		x
B. G. van Engelen	Radboudumc Nijmegen, Nl Baziel.vanEngelen@radboudumc.nl	Author			x	x
G. W. Padberg	Radboudumc Nijmegen, Nl georgepadberg@gmail.com	Author			x	x
S. Sacconi	Nice University Hospital, F sacconi.s@chu-nice.fr	Author		x		x
R. Lemmers	Leiden university medical centre, Nl R.J.L.F.Lemmers@lumc.nl	Author	x	x	x	x
S. van der Maarel	Leiden university medical centre, Nl S.M.van_der_Maarel@lumc.nl	Author	x	x	x	x
B. Eymard	Institute Myologie, Paris, F bruno.eymard@aphp.fr	Author	x	x	x	x
G. Bassez	Institute Myologie, Paris, F guillaume.bassez@aphp.fr	Author	x	x	x	x

*DoS* design or conceptualization of the study, *AoD* major role in the acquisition of data, *IoD* analysis or interpretation of the data, *DoM* drafting or revising the manuscript for intellectual content
