# Supplementary figures and images for: Scapular dyskinesis in myotonic dystrophy type 1: clinical characteristics and genetic investigations
Source: J Neurol. 2019 Aug 31;266(12):2987–96. doi: 10.1007/s00415-019-09494-8 (PMC6851043; doi:10.1007/s00415-019-09494-8)

## Slide 1
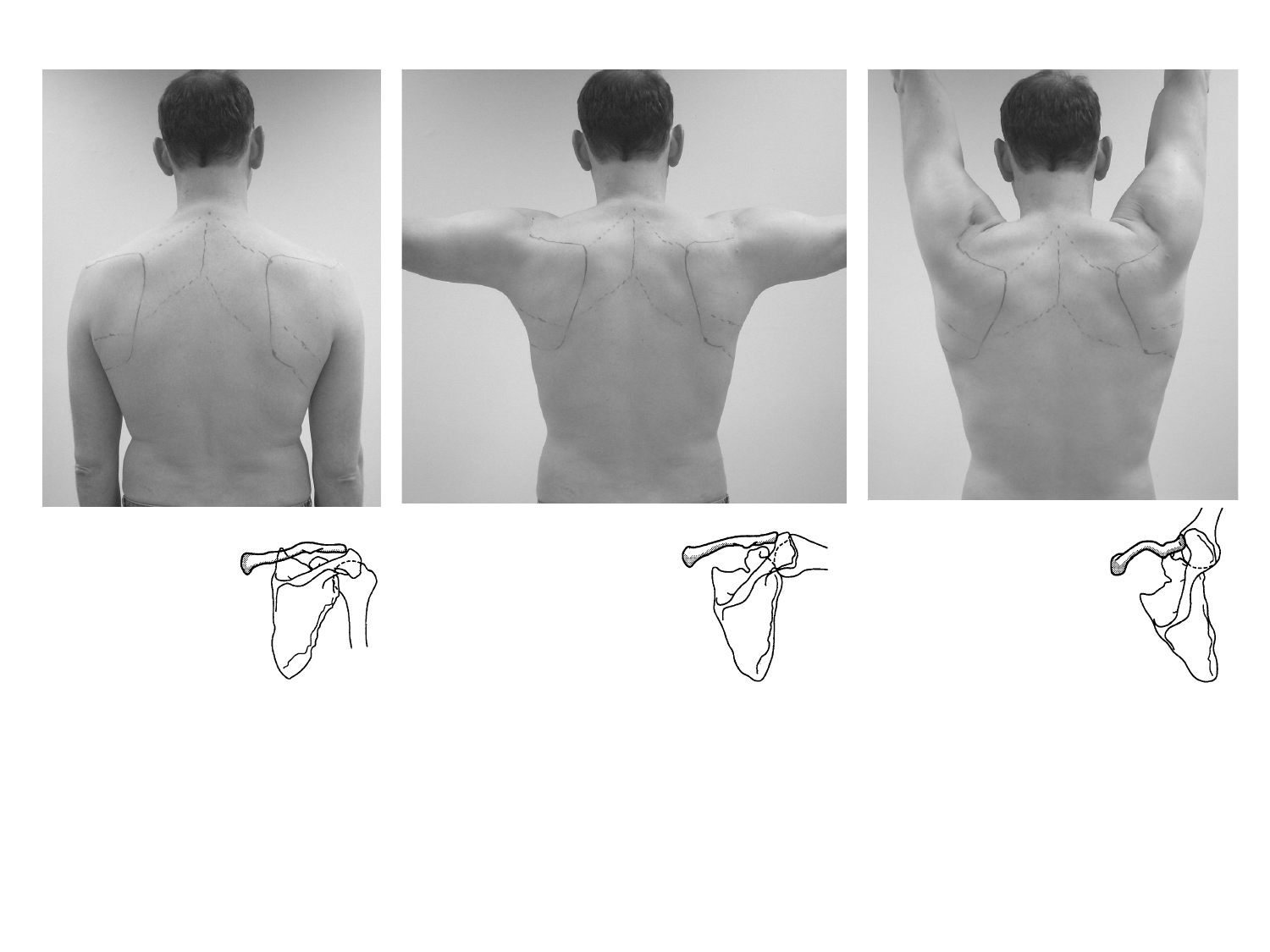

## Slide 2
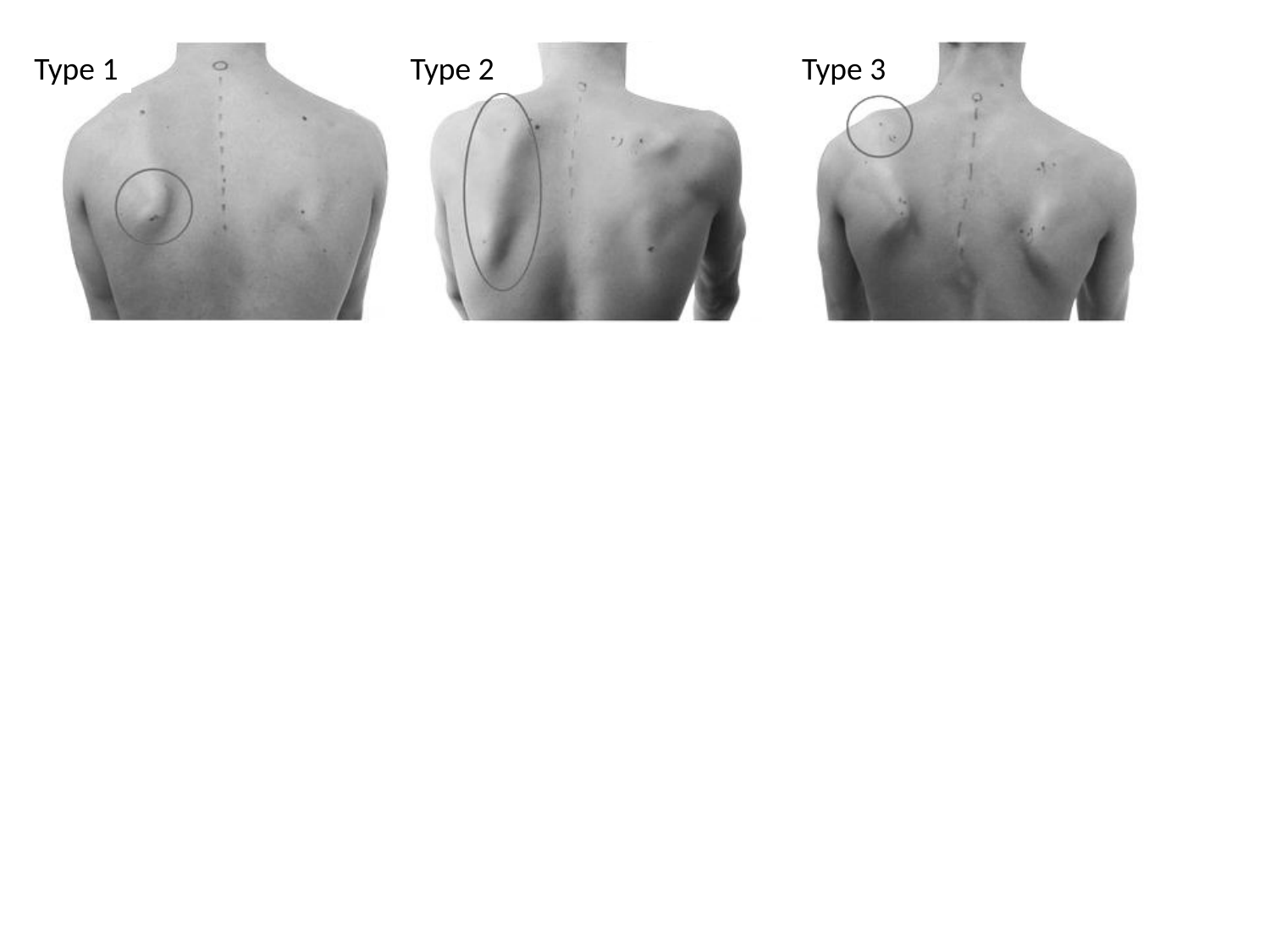

Type 1
Type 2
Type 2
Type 3

Supplement: Supplementary file 1 — Supplementary material 1 (PPTX 2036 kb) [file 415_2019_9494_MOESM1_ESM.pptx]
